# Understanding the shift to compulsion in addiction: insights from personality traits, social factors, and neurobiology

**DOI:** 10.3389/fpsyg.2024.1416222

**Published:** 2024-09-09

**Authors:** Haodong Su, Tongtong Ye, Songyan Cao, Chunyan Hu

**Affiliations:** ^1^College of Humanities, Anhui Science and Technology University, Chuzhou, China; ^2^Psychological Education Research Department, Anhui Science and Technology University, Chuzhou, China

**Keywords:** sign-tracker, goal-tracker, drug addiction, individual differences, compulsive characteristics

## Abstract

Compulsion stands as a central symptom of drug addiction; however, only a small fraction of individuals who use drugs exhibit compulsive characteristics. Differences observed in Sign-trackers (ST) and Goal-trackers (GT) during Pavlovian conditioning may shed light on individual variances in drug addiction. Here, we focus on the behavioral attributes, formation processes, and neural mechanisms underlying ST and how they drive addiction toward compulsivity in humans. We will explore addiction from three interconnected levels: individual personality traits, social factors, and neurobiology. Furthermore, we distinguish between the processes of sensitization and habituation within ST. These nuanced distinctions across various aspects of addiction will contribute to our understanding of the addiction development process and the formulation of targeted preventive strategies.

## 1 Introduction

Drug addiction represents a pressing global challenge in contemporary society, with approximately 243 million people worldwide grappling with substance abuse. This issue is accompanied by escalating societal costs, including increased healthcare expenditures, diminished productivity, and a surge in crime rates (UNODC, 2017). The core symptom of drug addiction lies in compulsive drug use behavior, where individuals persistently seek and consume drugs despite severe negative consequences (Zou et al., 2017).

However, a key issue in addiction is that not everyone transitions from recreational, controlled drug use to uncontrolled, compulsive drug use. Only a small minority, approximately 15–20% of individuals, cannot flexibly adjust their behavior (Anthony et al., 1994). This implies that there are significant individual differences in the process of transitioning toward addiction (Volkow and Morales, 2015). Susceptibility factors propel individuals from initial drug use to maintenance and the development of addiction. Recognizing these susceptibility factors is crucial for addiction prevention (Moggi, 2018).

In the classical Pavlovian conditioning paradigm, the identification of Sign-tracker (ST) and Goal-tracker (GT) as two distinct phenotypes provides crucial insights into understanding the driving forces behind individual susceptibility to addiction. Notably, these phenotypes may reflect inter-individual variations outlined in the incentive-sensitization theory. Specifically, these differences manifest as follows: (1) behaviorally, ST exhibits weakened inhibitory control, characterized by heightened novelty seeking and impulsivity traits (Flagel et al., 2014; Pitchers et al., 2017). A synthesis of previous literature suggests that these traits may be linked to two sequential stages in the transition to compulsivity; (2) Early negative experiences in ST, compared to GT, may be linked to the development of externalizing disorders (Flagel et al., 2009); (3) A comprehensive review of the literature suggests that the development of ST may share common neurobiological underpinnings with drug use. Given these points, it is imperative to incorporate the limited literature into a comprehensive framework encompassing psychological, social, and neurobiological factors. In this manuscript, we specifically review the unique aspects of ST across psychological, social, and neural domains, elucidating the driving roles of these three factors in the addiction process. Notably, we provide a more precise differentiation between the neural underpinnings of sensitization and habituation in addiction within the current manuscript.

## 2 The incentive-sensitization theory: sign-tracker versus goal-tracker

While consensus on the formation process of drug addiction remains elusive, the discourse has given rise to two classical theories, namely, the opponent process theory and the incentive-sensitization theory. The opponent-process theory posits that individuals initially experience the pleasurable effects of drug use (positive reinforcement). However, when individuals experience withdrawal symptoms such as tolerance, anxiety, and negative emotions, continued drug use alleviates these symptoms (negative reinforcement) (Solomon and Corbit, 1974). In summary, the opponent-process theory suggests that addiction involves drug choices driven by negative states. However, this theory does not account for individual differences in addiction and cannot explain why individuals may exhibit strong drug motivations even when they are not in a withdrawal state (Kalivas and McFarland, 2003). The incentive-sensitization theory provides a reasonable explanation for individual differences in addiction. According to this theory, addictive substances induce adaptive changes in the nervous system. These changes do not alter the pleasurable experience of the drug (“liking” the drug) but instead grant drug-related cues a strong motivational significance (“wanting” the drug). The incentive-sensitization theory successfully explains the separation of actions and intentions reported by many individuals with addiction and the fact that even after extended periods of abstinence, subtle environmental cues can trigger intense drug cravings (Hogarth and Hardy, 2017; Szumlinski et al., 2005). Individual differences in addiction stem from varying attributions, with the core characteristic of individual addiction being the attribution of reward to drug-related cues (Robinson and Berridge, 2000).

**Figure 1:**
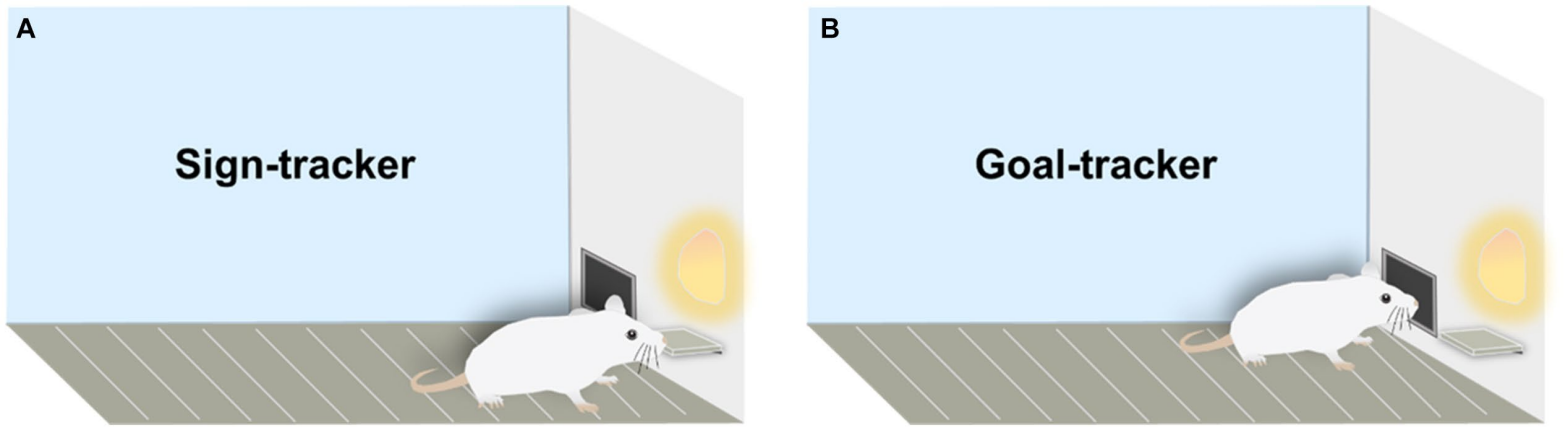
Sign-trackers and goal-trackers in rodents. **(A)** Sign-tracker: responsive to cues, rats display an interest in the lever, akin to the US (food), by approaching and nibbling on the lever. **(B)** Goal-tracker: focused on the goal, these rats show no interest in the cues and consistently approach the reward immediately after cue presentation.

Cues in the environment (e.g., lights, sounds, and odors) play a crucial role in Pavlovian conditioned approach (PCA) because they can predict positive or negative outcomes. In PCA, a neutral stimulus (e.g., lever-cue) paired repeatedly with an unconditioned stimulus (US, e.g., food) becomes a conditioned stimulus (CS), with the CS always preceding the US. Subsequently, the rat develops a conditioned response (CR) to the CS (Pavlov, 1927). While it might seem intuitive that rats would be more interested in the food that eventually drops than in the unremarkable lever-cue, this is not the case. Based on different lever-approach patterns in the PCA scenario, two distinct phenotypes—sign-tracker and goal-tracker—have been identified. For GT, the lever-cue is merely a predictor, eliciting a conditioned response directed at the location of reward delivery. However, for ST, the lever-cue is endowed with both predictive and incentive value (Boakes, 1977; Hearst and Jenkins, 1974). Therefore, GT, as goal responders, are less sensitive to cues predicting rewards. For instance, they may initially approach the food trough when it suggests a reward is available and do not invest as much time and effort in lever (Robinson and Flagel, 2009). On the other hand, ST are cue responders in PCA, with reward-predictive cues holding greater incentive salience for them. For example, they first approach the lever, and this incentive value of the cue persists even in the absence of the US (Figure 1) (Flagel and Robinson, 2017).

The Pavlovian–instrumental transfer (PIT) paradigm is used to explore how stimuli acquired through classical conditioning (e.g., a bell associated with food) influence behavior in operant conditioning tasks (e.g., pressing a lever to obtain a food reward). For instance, during the PIT testing phase, the CS from the classical conditioning stage (e.g., the bell) is presented without the food reward, while the rat’s lever-pressing behavior is observed. Notably, in this phase, lever presses are no longer reinforced with food rewards (Estes, 1943). Research has shown that ST exhibit a stronger PIT effect compared to GT, meaning that ST continue to engage in vigorous lever-pressing behavior even in the absence of a food reward (Corbit and Balleine, 2011). This suggests that the CS holds significant incentive value for ST during the PIT test.

Overall, the ST and GT phenotypes identified in the PCA, along with the strong PIT effect observed in ST, align perfectly with the incentive-sensitization theory. As proposed by this theory, pathological incentive motivation toward cues may be a driving factor in addiction. Accordingly, research has reported that ST are more likely than GT to predict compulsive drug-use behavior. The 3-CRIT criteria are used to assess compulsive drug use in animal models. These criteria include: (1) resistance to punishment (such as shock) during continued drug responses, (2) continued responses (drug craving) when the drug is unavailable, and (3) motivation to seek the drug under progressive ratio schedules (Deroche-Gamonet et al., 2004; Spanagel, 2017). Research findings indeed suggest that when subjected to relevant tests, ST individuals exhibit features of compulsion as outlined in the 3-CRIT criteria (Fitzpatrick et al., 2019; Morrison et al., 2015; Saunders and Robinson, 2011). Despite reports of contradictory results, when the CS is devalued (a previously reward-paired CS is now paired with an aversive stimulus, such as lithium chloride), ST individuals do exhibit reduced responses to CS. However, this may be context-dependent, as ST can lose sensitivity to CS devaluation when the contextual environment is inconsistent, subsequently reverting to compulsive seeking (Amaya et al., 2020; Derman et al., 2018). This aligns with real-world scenarios, such as the effectiveness of addiction treatment in controlled settings but susceptibility to cue-induced relapse in daily life. Furthermore, ST and drug abuse share common neural foundation (Tomie et al., 2008). Thus, the potential differences between ST and GT phenotypes may indeed be related to individual variations in addiction.

## 3 Mapping sign-tracking and goal-tracking onto human behaviors

**Figure 2:**
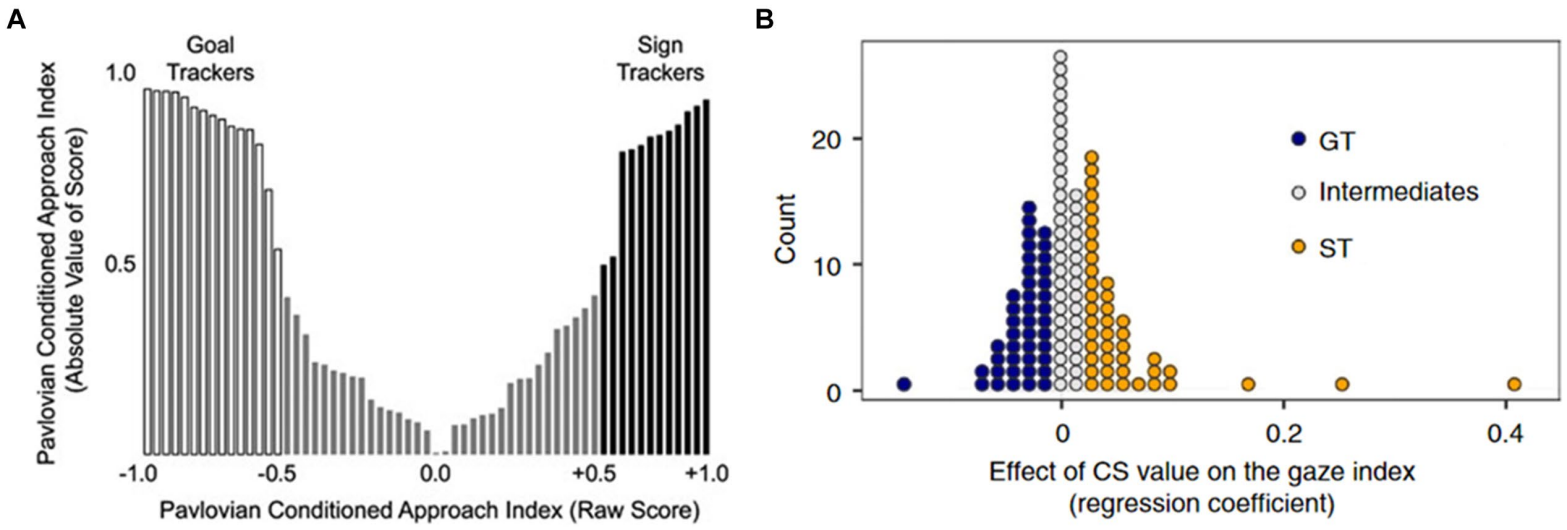
Distribution of sign-trackers and goal-trackers in human and animal studies. **(A)** In rodents, sign-trackers and goal-trackers exhibit a nearly symmetrical distribution (Saunders and Robinson, 2011). **(B)** In human studies, sign-trackers and goal-trackers display a proportion similar to that observed in animal research (Schad et al., 2019).

Another pivotal question revolves around whether these two phenotypes observed in animal models exhibit consistent or analogous patterns in humans. This consideration affects the potential for the susceptibility demonstrated within the ST phenotype to hold relevance for human translation. Previous research suggests that such a translation is not only possible but also reasonable. In human studies, akin to findings in animal models, both ST and GT phenotypes have been identified (Schad et al., 2019). While intermediate types also exist (in line with animal models), a predominant bimodal distribution trend has emerged in human populations (Figure 2). Moreover, investigations employing eye-tracking and functional magnetic resonance imaging (fMRI) techniques have provided evidence of distinct neural mechanisms for ST and GT in humans (Schad et al., 2019). Some researchers have commented on the applicability of analogizing ST traits to humans (Colaizzi et al., 2020). Similarly, studies have indicated that in humans, individuals who exhibit a focus on cues akin to ST may also be associated with more severe addiction and compulsivity (Albertella et al., 2019; Colaizzi et al., 2020). Given our previous discussion, it is appropriate to translate the behavioral phenotypes of ST and GT observed in animal models to human studies. Moreover, the behavioral characteristics exhibited by ST provide valuable insights into understanding the susceptibility factors underlying human addiction. There is increasing evidence that substance addiction is associated with habitual behavioral patterns (Lloyd et al., 2014; Sjoerds et al., 2013; Vollstädt-Klein et al., 2010; Zapata et al., 2010). Second-order schedules can be used to investigate drug-related cue-reinforced seeking responses, such as studying the transition from goal-directed to habitual seeking controlled by CS in substances like heroin, cocaine, and alcohol (Di Ciano and Everitt, 2005; Everitt and Robbins, 2000; Giuliano et al., 2017). However, there is ongoing debate about whether habitual behaviors observed in laboratory settings (in both animal and human models) fully capture all the characteristics of habits as they occur in everyday human life (Pool et al., 2022). Nevertheless, the core features of both do show significant similarities (Dickinson and Pérez, 2018; Everitt et al., 2008). This debate may partly stem from the inconsistent definitions of habits used across different studies (De Houwer et al., 2023). Therefore, we will refrain from delving too deeply into this issue here.

## 4 Sensitization and habituation of cues: a psychological and neurobiological separation

Current research often conflates cue incentive sensitization and habituation within ST as the same process. This review argues that these two phenomena are not equivalent and are distinct in both psychological processes and neural foundations. Sensitization refers to the excessive amplification of psychological “wanting,” especially when triggered by cues (Berridge and Robinson, 2016). The term “wanting” is placed in quotes to denote a specific type of desire, one that is typically elicited by cues associated with rewards or by vivid imagery of the reward itself (Berridge, 2012). Sensitization implies that these cues possess certain characteristics: (1) they capture and bias attention, and (2) they trigger behaviors aimed at acquiring and seeking rewards (Anderson and Yantis, 2013; Peciña and Berridge, 2013). On the other hand, habituation is described as the process by which the reinforcing properties of a drug diminish with continued use, leading individuals to transition into a behavior pattern that is less flexible and goal-directed, and more driven by stimuli (Everitt and Robbins, 2005).

This indicates that cue sensitization in ST remains goal-directed behavior, determined by the high incentive value of cues. This excessive attention to cues may be related to attentional bias and reflect poor attentional control in individuals. Its neural basis may be associated with dopamine neurons projecting from the ventral tegmental area (VTA) and substantia nigra compacta (SNc) to forebrain targets like the nucleus accumbens (NAc), encoding motivational behaviors (Berridge and Robinson, 2016; Budygin et al., 2020; Koshy Cherian et al., 2017; Kutlu et al., 2022). The habituation within ST is related to a weakening of behavioral inhibition in individuals, reflecting a lack of planning in behavior. For example, compared to GT, ST individuals exhibit earlier and more frequent lever-pressing behavior (Flagel et al., 2009). This pattern of weakened inhibition primarily manifests as difficulties in top-down control, potentially associated with deficits in cholinergic modulation in the cortex, the gradual waning of cortical control over subcortical structures, and the shift from the ventral to dorsal striatum [from ventral striatum (VTS) and dorsal medial striatum (DMS) to dorsal lateral striatum (DLS)]. It is worth noting the synaptic plasticity between the cortex and striatum, which is linked to the Dopaminergic neuronal projection from the midbrain to nucleus accumbens medium spiny neurons (MSN) (Barker et al., 2015; Giuliano et al., 2019; Pascoli et al., 2014; Pascoli et al., 2011; Sarter and Phillips, 2018). This further emphasizes that learning associated with high-incentive cue associations may form the foundation for the transition to habitual behaviors. The failure of goal-directed control and the dominance of habitual behavior patterns may serve as the basis for the shift from controlled drug use to compulsive drug use (Lüscher et al., 2020).

Hence, the behavioral characteristics, underlying factors, and biological basis exhibited by ST seem to shed light on individual differences in human addiction. First, concerning behavioral characteristics, as mentioned earlier, ST individuals are more prone to cue sensitization, which may be associated with attentional biases. Conversely, habituation may be linked to poorer behavioral inhibition, a trait often found in individuals with impulsivity (Pattij and De Vries, 2013). Individuals with high impulsivity traits often exhibit reduced attentional capacity and behavioral inhibition, which has been associated with impulsive behaviors and various substance use disorders in human studies (Hildebrandt et al., 2021; Potvin et al., 2018). In the laboratory, different behavioral paradigms can be used to assess various aspects of impulsivity. For example, the delay discounting task is used to measure choice impulsivity, reflecting an individual’s tendency to devalue delayed rewards and make high-risk decisions. The Stop-Signal Task measures response inhibition, referring to the inability to control actions and effectively halt initiated behaviors (MacKillop et al., 2016). Notably, there is no difference between ST and GT in impulsive choice, suggesting that ST may specifically struggle with behavioral inhibition, rather than being sensitive to issues of temporal or probabilistic discounting (Flagel et al., 2009).

Furthermore, the use of selectively bred High-Responder (BHR) and Low-Responder (LHR) rats has shown differences in incentive attribution, with BHR primarily showing ST characteristics. This implies that novelty-seeking traits may be susceptibility factors for addiction (Flagel et al., 2014). There is already considerable evidence linking novelty-seeking personality traits to substance addiction, including nicotine and cocaine (Belin and Deroche-Gamonet, 2012; Perkins et al., 2008). Secondly, the driving role of social factors in ST is noteworthy. In humans, substance abuse or addiction is often associated with a range of other behavioral syndromes collectively referred to as “externalizing disorders,” which includes impulsivity (Krueger et al., 2007). These “externalizing disorders” are linked to early-life environmental stress, developmental experiences, and attachment relationships (typically with caregivers), with positive parenting being a protective factor against these disorders (Buu et al., 2009; Otten et al., 2019; Wakeford et al., 2018). In summary, this review comprehensively examines the factors reflected in sign-tracking (ST) behavior, including personality traits, social influences, and neurobiological mechanisms, and their roles in driving uncontrolled drug use in individuals.

## 5 Compulsivity driven by personality traits

### 5.1 Novelty seeking

Novelty seeking refers to the tendency to initiate behavior in response to new stimuli and potential rewards. This trait was initially introduced as part of the biopsychosocial model proposed by Cloninger (1993). This model is based on complex interactions among genetics, psychology, social influences, culture, and spiritual dimensions, categorizing an individual’s personality traits into two major aspects: temperament and character, consisting of a total of seven sub-dimensions (Cloninger, 1993). Cloninger posited that novelty seeking is a component of an individual’s temperament module, representing a non-learned instinctual behavior characterized by a high motivation for new stimuli in the environment (Beckmann et al., 2011). As mentioned earlier, selectively bred rats that exhibit different responses to novel stimuli also show differences in attribution to incentives. Rats with high novelty-seeking tendencies often display ST in PCA, suggesting that the novelty-seeking trait might be one of the susceptibility factors in the transition to compulsive behaviors (Flagel et al., 2014).

However, research on novelty seeking in addiction has yielded mixed results. In human studies, the novelty-seeking trait can predict susceptibility in the initial stages of self-administration and compulsive drug use. Novelty-seeking levels measured in early adulthood can serve as predictive factors for the abuse of substances such as alcohol, nicotine, cannabis, and various other substances (Belin and Deroche-Gamonet, 2012; Foulds et al., 2017; Stautz and Cooper, 2013). But another animal study that aligns with the 3-CRIT suggested that the high novelty-seeking phenotype cannot predict binge-like drinking behavior in mice (Radwanska and Kaczmarek, 2011). It’s worth noting that Belin et al.’s research may reveal a complex structure of the novelty-seeking trait, with its different dimensions being related to different aspects of addiction (Belin and Deroche-Gamonet, 2012). As defined by Zuckerman et al. (1993), novelty seeking is “a personality trait characterized by a tendency to actively seek out new and exciting sensations, and a willingness to take physical, social, legal, and other risks for the sake of experiencing these novel sensations.” Questionnaire measurements of novelty-seeking traits in human studies also encompass dimensions such as impulsivity, exploratory excitement, and disorderliness (Garcia et al., 2017).

Therefore, novelty-seeking measured in animal models may not capture all the features of human novelty-seeking, which could be a reason for the inconsistencies between human and animal research findings. This notion is supported by studies that investigate subtypes of novelty-seeking. Researchers have further subdivided high novelty-preferring rats (BHR) into high novelty preference (HNP) and low novelty preference (LNP) subtypes based on their choice preference in free-choice procedures. The results suggest that HNP may be a susceptibility factor for compulsive cocaine use, promoting the transition from cocaine use to compulsive behavior (Belin et al., 2010). These findings suggest the existence of novelty-seeking subtypes in animals that may be analogous to those in humans. Employing more refined behavioral paradigms could enhance our understanding of the mechanisms underlying compulsive drug use in addiction.

Novelty-seeking may predict the transition from drug use to compulsive behavior due to two potential factors. From a neural mechanism perspective, novelty-seeking and drug-using may share a common biological basis. For example, many addictive drugs lead to an increase in dopamine levels in the mesolimbic system. Alcohol has complex effects on gamma-aminobutyric acid (GABA) and glutamate receptors, resulting in rapid changes in dopamine levels in the NAc (Abrahao et al., 2017). Cocaine, as a potent stimulant, increases dopamine levels by blocking the reuptake of dopamine at neuronal terminals, while nicotine can directly depolarize dopamine neurons (Lüscher and Ungless, 2006). Among the neurotransmitters associated with substance addiction, some have also been reported in studies related to novelty-seeking behavior. For example, Rohan et al. (2021) revealed that exposure to a new environment could activate neural pathways shared with addiction. Behaviorally, novelty-seeking may encompass characteristics such as poor attention and impulsivity, all of which fall under the concept of behavioral inhibition. These traits could be significant driving forces behind the compulsive cue-seeking behavior observed in ST.

### 5.2 The trait of impulsivity

The trait of impulsivity is a complex, multidimensional construct that can conceptually be divided into two main components: impulsive behavior and impulsive choice. High impulsivity has been associated with a range of psychiatric disorders, including bipolar disorder, attention-deficit/hyperactivity disorder, and borderline personality disorder, among others (Bayes et al., 2019; Berg et al., 2015). Within the realm of personality traits, impulsivity is generally defined as “the tendency to make rapid, unplanned, or reward-driven responses to internal or external stimuli without adequately considering the potential consequences for oneself and others” (Moeller et al., 2001). The description of the impulsivity trait in humans aligns with the characteristics of ST. As previously discussed, we distinguished between sensitization and habituation in the formation of ST behavior, which is consistent with the attention deficits and lack of planning features observed in human impulsivity traits.

Firstly, there’s sensitization. ST attributes the incentive value to cues rather than drugs, and even when the reward is lost (e.g., food), it cannot stop the attention to cues. For example, raccoons may become fixated on biting coins (US) and miss out on food (CS) rewards (Breland and Breland, 1961; Bucker and Theeuwes, 2016). Attention control deficits may be related to this behavioral pattern. For example, ST typically perform poorly in sustained attention tasks (SAT) (Pitchers et al., 2017). The attention capture related to cue rewards may form the basis for incentive attribution, and this cue sensitization may predict compulsive behavior in addiction. In human studies, cue-reward-related attention capture has been found to predict an individual’s addiction and compulsive behavior, and is associated with the severity of compulsive behavior. This attention bias may be the basis for cognitive inflexibility patterns (Albertella et al., 2019; Albertella et al., 2020).

**Figure 3:**
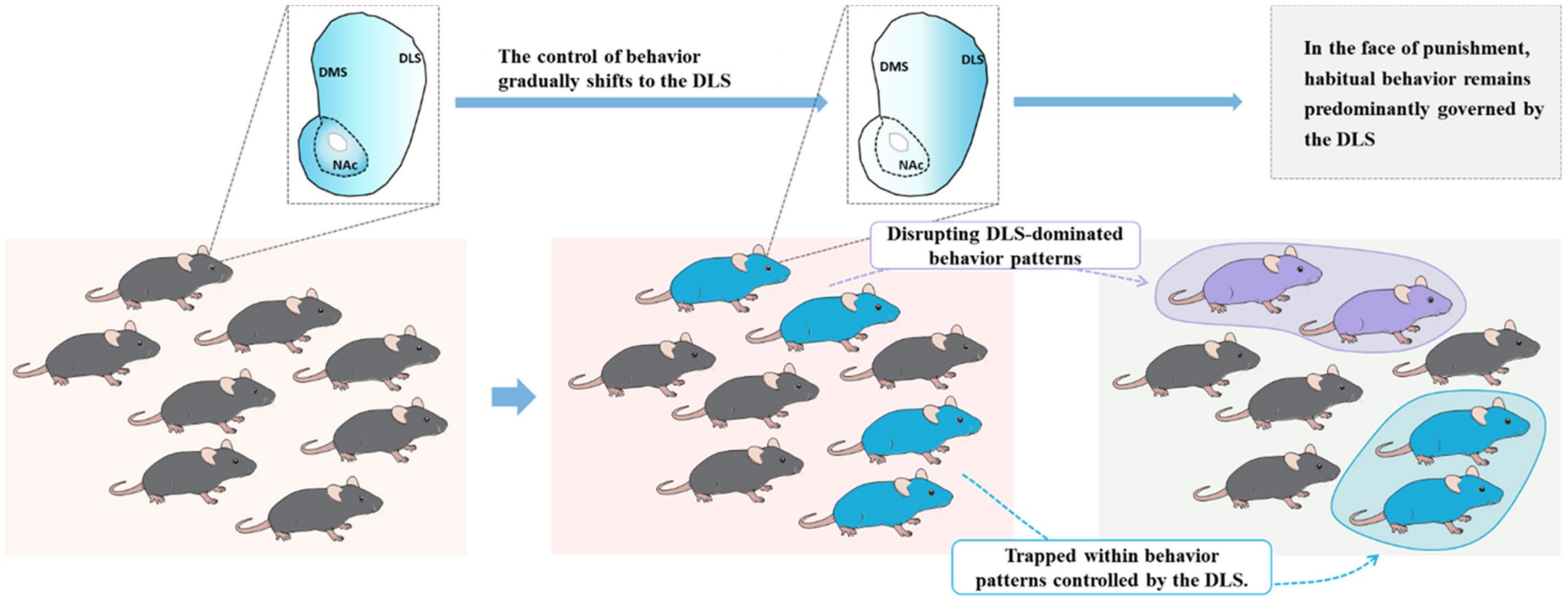
Failure to disengage from the dorsolateral striatum (DLS) reflects the compulsivity of addiction. In native rats, individuals typically maintain a balance between goal-directed and habitual behaviors. However, during drug exposure, those individuals dominated by the DLS gradually transition toward compulsivity.

Compulsive behavior can be understood as a focus on the immediate action despite adverse consequences, losing the association between behavior and consequences. Attentional narrowing may be a precursor to transitioning to these compulsive traits, but at this stage, it is still goal-oriented (Brown et al., 2018). Therefore, the core features of compulsive use in addiction are the excessive habitual behavior (lack of planning) following cue sensitization and the inability to break free from habit-based control during drug use (Figure 3). Habitual behavior is based on stimulus–response associations and typically occurs after extensive training. Once habits are established, they require fewer cognitive resources, making the response often independent of outcome value, triggered by specific cues or stimuli (automatic attention to cues) (Dickinson, 1985). The reduced capacity for top-down behavioral inhibition observed in ST may make it more prone to habit formation. In other words, in the competition between goal-directed and habitual behavioral patterns, ST individuals may be more inclined to have their behavior dominated by habit-based patterns (Campus et al., 2019).

Therefore, the impulsivity trait may link habit formation with compulsive drug-taking behavior. It is this deficient top-down control that makes it difficult to quit drug use and shift back to goal-directed behavior. Normally, when there is reward devaluation, individuals quickly revert to goal-directed behavior. However, individuals with high impulsivity traits, due to poor attention control and weakened top-down behavioral inhibition, are prone to remain sensitized to cues, maintaining habitual attention to cues and subsequent behavioral responses. In drug use, this habit-dominated behavioral pattern leads to the transition to compulsive drug-taking and eventually evolves into uncontrolled drug-seeking behavior.

## 6 Social factors in addiction

High impulsivity and addiction both fall under the category of externalizing disorders, and the development of such externalizing disorders is influenced by an individual’s early life experiences. Therefore, to further understand the origins of impulsivity, attention deficits, and novelty-seeking behavior, we will delve into the psychodynamic perspective to comprehend the psychological processes involved in the transition toward compulsive drug use during an individual’s drug use development. Adverse experiences, the establishment of attachment relationships, and exposure to stress during an individual’s early developmental stages may influence susceptibility to compulsive behavior in addiction (Alvarez-Monjaras et al., 2018). It’s important to note that these adverse experiences, attachment, and stress are not isolated factors; they often interconnect and mutually affect each other. For instance, early separation from caregivers during childhood is both an adverse experience and an example of an insecure attachment relationship. While these factors overlap, they also have distinctions. For instance, early adverse experiences may be related to the dopamine neural system, attachment relationships emphasize social interactions and may be related to the oxytocin system, and stress may be associated with an individual’s hypothalamic–pituitary–adrenal (HPA) axis function (Kim et al., 2016).

### 6.1 Early adverse experiences

Early adverse experiences in rodents are typically characterized by disrupted caregiving behaviors, such as premature separation from the mother. Research has shown that animals experiencing such adverse experiences tend to exhibit high novelty-seeking behaviors in adulthood, consistent with what was mentioned earlier. In addition to showing high seeking traits, they also demonstrate greater sensitivity to addictive substances (Brake et al., 2004; Kim et al., 2016; Zimmerberg and Shartrand, 2004). These early adverse experiences may physiologically impact the activity of dopamine neurons in the brain. For instance, adolescent rats exposed to early maternal separation exhibit alterations in baseline dopamine levels in the striatum and prefrontal cortex (Llorente et al., 2010). Importantly, there are also changes in the levels of dopamine release induced by stimuli. For instance, an enhanced dopamine release in response to rewarding stimuli has been observed in the VST and hypothalamus (Arborelius and Eklund, 2007). The altered patterns of reward and motivation-related brain dopamine neuron activity resulting from these early adverse experiences have also been consistently observed in human research (Pruessner et al., 2004). These changes in dopamine neuron activity patterns may be related to the attribution of incentive to subsequent drug-related cues (Colaizzi et al., 2023; Hynes et al., 2018). Additionally, early adverse experiences can lead to the development of compulsive behavioral traits during adolescence (Brydges et al., 2015). All of these findings suggest that early adverse experiences in individuals may drive drug use behaviors in addiction toward compulsivity.

### 6.2 Attachment relationships

Secure attachment relationships have a protective effect against substance abuse in adulthood (Tomie, 2018). The positive social interactions between individuals and their caregivers contribute to the development of executive functions and self-regulation. In contrast, a lack of soothing and positive attachment experiences can hinder the establishment of these functions, eventually manifesting as impulsive traits, particularly attention deficits and weakened behavioral inhibition within an individual’s personality. For instance, rodent models have demonstrated that rats with insufficient early social interaction experiences (poor attachment experiences) exhibit significant arousal toward reward-related cues, leading to a loss of behavioral inhibition (Lomanowska et al., 2011). On the other hand, the HPA axis is believed to directly influence the behavior of ST and GT within the PCA, with ST showing greater cortisol release during single PCA sessions (Beckmann and Bardo, 2012; Tomie et al., 2000). However, positive social interaction experiences can reduce HPA axis activity, inhibiting ST’s seeking responses to cues within the PCA (Beckmann and Bardo, 2012). Recent research suggests that attachment and addiction may share a common neural basis. Therefore, individuals with maladaptive attachment relationships may have their neural systems perpetuating the development of addictive behaviors (Burkett and Young, 2012; Johns et al., 2011).

### 6.3 Stress

Early life stress events can lead to maladaptive tendencies, such as children facing extremely harsh parenting styles often exhibiting higher levels of impulse control disorders and externalizing disorders (Vasiou et al., 2023). In rodent studies, rats subjected to stress due to social isolation exhibited more cue-induced sensitization characteristics and showed heightened locomotor reactivity to novel stimuli during adolescence (Baarendse et al., 2013; Lomanowska et al., 2011). Furthermore, the HPA axis is involved in a series of cascading neurotransmitter and hormone regulatory processes related to stress. In chronic stress environments, sustained activation of the HPA axis can enhance extracellular DA release in the striatum, indirectly impacting neural pathways encoding motivation and reward within the brain (Nikulina et al., 2012; Wang et al., 2005). Overall, these findings suggest that early life stress events may shape susceptibility traits for compulsive behavior in addiction.

In summary, whether it’s attachment relationships or stressful events, their influence on individuals during early life is enduring and subtle, and this developmental social factors always impacts inhibitory functions behaviorally. Physiologically, it is invariably associated with the neural foundations that encode motivation and goal-directed or habitual behaviors. From a psychological process perspective, it indeed drives the formation of impulsive behaviors, laying the foundation for the transition of drug use in addiction toward compulsivity.

## 7 Neurobiology of the transition to compulsion

### 7.1 Neural basis of cue sensitization

In the preceding sections, we have discussed how personality traits and social factors drive the transition from drug use to compulsive drug use. However, another crucial question is how the brain’s relevant neural systems participate in the transition to addiction. First and foremost is the sensitization to cues, where the encoding of cues in a highly motivated state may rely on the phasic release of dopamine in the NAc. The use of fast-scan cyclic voltammetry allows for the measurement of dopamine changes at a sub-second timescale. The results indicate that differences in dopamine responses between reward and prediction occur only in the ST system, with no significant changes observed in the GT system (Flagel et al., 2010). This suggests that the phasic changes in dopamine in the NAc may not encode the traditional “reward prediction error hypothesis,” which relates to the encoding of prediction and actual reward discrepancies. Instead, it may encode the incentive value of cues and is mediated by dopamine in the Nucleus Accumbens core (NAcc) (Dalley et al., 2005; Di Ciano et al., 2001; Montague et al., 1996). This proposition was further confirmed by systemic administration of flupenthixol, a non-selective dopamine antagonist, which showed that blocking dopamine had an impact on learning in the ST system within the PCA, while the GT system remained largely unaffected (Flagel et al., 2010).

Furthermore, the sustained incentive for cues in the ST system is also associated with the dopamine content in the NAcc. During cue-induced reinstatement tests, the ST system exhibits higher responsiveness compared to the GT system. Blocking dopamine in the NAcc can attenuate this response in the ST system, leading to behavior similar to that of the GT system (Saunders et al., 2013). The upregulation of the dopamine transporter (DAT) in the ST system may be related to the increased dopamine concentration in the Nucleus Accumbens core (NAcc) induced by AMPH use. When dopamine is released from neurons into the extracellular space, DAT on the synaptic surface plays a primary role in clearing and recycling excess dopamine. The longer dopamine stays in the extracellular space, the more it interacts with neighboring neurons, leading to dysregulation in the system (Singer et al., 2016). Therefore, compared to the GT system, dopamine reuptake in the extracellular space of the NAcc occurs more rapidly in the ST system. However, some addictive substances can increase synaptic dopamine levels by blocking and inhibiting DAT. For example, directly injecting amphetamine into the NAcc can slow down the dopamine reuptake process in the ST system (Singer et al., 2016). The synaptic dopamine that is not cleared primarily binds to D1 and D2 receptors and plays a significant role in encoding the incentive salience (Flagel et al., 2016). Furthermore, in Long-Evans rats, the high expression of DAT phenotype can predict cocaine-like addictive behaviors (Yamamoto et al., 2013). Therefore, these results suggest that the pattern of DA release in the NAcc may serve as the neural basis for ST’s attribution of incentive salience to cues, and this could also represent a susceptibility characteristic for the transition to compulsive drug-taking behavior.

The formation of the ST is therefore dependent on dopamine, and the pattern of dopamine release in the NACc is crucial for the reward learning process. The dopamine neurons in the NACc primarily originate from dense projections of the midbrain VTA and SNc, and this mesolimbic dopamine neural circuit plays a significant role in encoding cue-induced motivation (Volkow et al., 2017). It is noteworthy that dopamine neurons output from the VTA and SNc exchange information in the striatum through MSN and glutamatergic neurons from the cortex, forming a circuit with a spiral-like structure reminiscent of a serial loop (Haber et al., 2000). This also underscores that changes in the neural system sensitized to cue-induced stimulation will further impact the cortical structure and ventral striatum regulation of target behaviors.

Another crucial brain region related to cue-induced motivation is the hippocampus. The hippocampus is involved in various types of memory, especially context-related cues (Burgess et al., 2002; Martin and Clark, 2007). Anatomically, the hippocampus can be divided into the ventral hippocampus (VHipp) and the dorsal hippocampus (DHipp). VHipp projects glutamatergic neurons to the NAc and is thus potentially involved in cue-driven motivation through this pathway. For instance, it has been shown that damage to VHipp can impact the concentration of dopamine in the NAc and inhibit cue-seeking behaviors in the ST within the PCA (Fitzpatrick et al., 2016). In summary, the hippocampus, as a regulator of contextual or spatial stimuli, may play a crucial role in the motivational effects of cues on drug seeking (Selden et al., 1991).

The basolateral amygdala (BLA) also plays a crucial role in cue-induced motivation for drug seeking, and it is the BLA-NAc connectivity loop that is particularly essential. Selective damage to either the BLA or NAc, effectively disconnecting the two, has no impact on self-administration behavior for cocaine but impairs cocaine seeking in secondary reinforcement procedures (Di Ciano and Everitt, 2004). This suggests that there are distinctions at the neural circuit level between drug seeking and drug taking. In summary, maintaining cue-controlled drug seeking requires the involvement of the BLA-NAc connection, and this connection forms a serial loop circuit with the involvement of the prefrontal cortex and striatum.

### 7.2 The top-down loss of control and the transition to dorsal striatum control pattern

The projection of dopamine neurons from the midbrain to the striatum mediates the sensitization of cue-induced motivation, and these neural system’s plastic changes also lay the foundation for subsequent habitual behavioral patterns. However, the core of transitioning to compulsive seeking in addiction is the inability to break free from habit-dominated behavioral patterns. Therefore, the neural basis of habitual behavior in individuals may be related to the susceptibility mechanisms underlying compulsive drug use. In cue-induced motivation, an individual’s behavior is still in a state of balance between goal-directed and habitual actions. As mentioned earlier, within the ST, not only is there sensitization to cues but also changes driven by habitual behaviors. Unlike goal-directed behavior, this form of habit-driven behavior is considered a crucial foundation for the transition to compulsive seeking (Lüscher et al., 2020). This gradual transition to a habitual pattern of behavior, which remains resistant to punishment, may have its neural basis in the difficulty of top-down control from cortical regions to subcortical structures and the dominance of the DLS in behavior control (Furlong et al., 2014; Hyman and Malenka, 2001; Smith and Laiks, 2018).

It is widely recognized that the PFC plays a crucial role in maintaining goal-directed behavior (Friedman and Robbins, 2022). However, many addictive substances can impair PFC function. For example, in individuals with alcohol addiction, brain regions associated with goal-directed behavior (vmPFC and ventral striatum) have been found to be less active compared to control groups, while regions associated with habit (e.g., the nucleus accumbens shell, equivalent to the dorsolateral striatum in rodents) show increased activity (Sjoerds et al., 2013). Although this frontal lobe damage is related to substance intake, individual susceptibility factors may also play a role. Studies involving drug-addicted individuals and their non-addicted siblings have found that both groups exhibit impairments in frontal lobe function. High impulsivity traits are often associated with difficulties in frontal lobe-mediated behavioral inhibition, suggesting that impulsivity traits in personality may serve as susceptibility factors for addiction (Ersche et al., 2011; Ersche et al., 2012). This impairment of PFC function can also affect an individual’s executive functions and lead to decision-making deficits, which may, in turn, drive the development of addiction (Bechara et al., 2001). Impairments in executive functions can result in poor inhibition of habitual behaviors, thereby prioritizing the output of habitual behaviors in response to cues (Hardwick et al., 2019).

**Figure 4:**
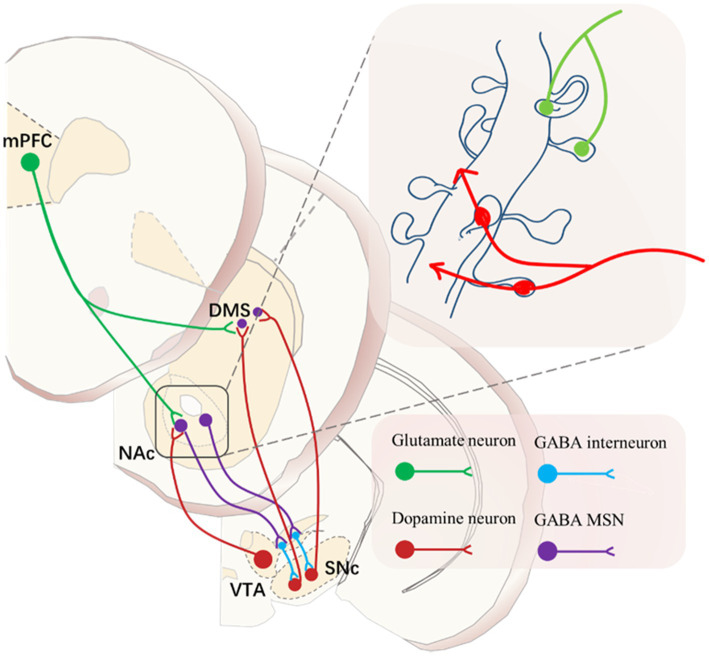
Predominant neuronal type in the NAc in the striatum, approximately 95% of neurons are of the Medium Spiny Neuron (MSN) type, characterized by GABA as their primary neurotransmitter. The dendritic spines on MSN neurons resemble antennae and serve as sites for information exchange between glutamatergic neurons from the cortex and dopaminergic neurons from the midbrain.

As the glutamatergic neurons from the prefrontal cortex interact with the dopamine neurons projecting from the VTA and SNc to the striatum in a serial loop circuit, the balance between goal-directed and habitual behavior patterns is likely disrupted, leading to the establishment of habit-dominated behavior patterns (Haber et al., 2000) (Figure 4). Previous research supports this, indicating that top-down cortical control mechanisms are intact in GT but may be impaired in ST (Campus et al., 2019; Haight et al., 2017). Consequently, the shift in control from the ventral to the dorsal striatum might suggest the neural basis for the transition to compulsive drug use.

The important roles of the DMS and DLS in compulsive drug use have been confirmed in both human and animal research. For example, the anterior part of the DLS (aDLS) plays a prominent role in the transition to compulsive seeking; functional magnetic resonance imaging (fMRI) studies in humans have shown increased activation in the DMS when individuals who engage in recreational drug use see drug-related cues, while addicted individuals show enhanced DLS activity (Everitt and Robbins, 2005, 2016; Zhou et al., 2019). The “seeking-taking” chained procedure distinguishes compulsive seeking and taking of drugs. In this paradigm, animals must perform an action (such as pressing a lever) to “seek” another task that allows them to “take” the drug. After several weeks of training, a shift in control from the DMS to the DLS was observed in animals that were insensitive to reinforcement devaluation (Corbit et al., 2012). This indicates a shift from goal-directed to habit-dominated behavior patterns in this paradigm. Furthermore, in the same training procedure, when rats exhibited habit-dominated behavior, inhibiting DLS activity forced the habit system offline, and rats again exhibited sensitivity to reinforcement devaluation (Zapata et al., 2010). In summary, these results suggest that in the transition to compulsive seeking, seeking behavior dependent on the DMS gradually becomes dominated by habitual seeking responses dependent on DLS activity.

Therefore, as it stands, the significant individual differences observed in addiction are not only related to sensitization to cues during drug use but, more importantly, to an overreliance on the DLS during the drug use process and an inability to break free from habit-dominated behavior driven by the DLS. When faced with punishment, individuals who cannot disengage from the DLS will exhibit characteristics of compulsive drug use (Figure 3). The dopaminergic neurons projecting from the VTA to the NACc are associated with GABAergic MSN in the NAC region. GABAergic MSN neurons project back to subcortical areas, inhibiting dopamine neurons located outside the VTA. These dopamine neurons subsequently project to the NAC shell. This circuit forms a feedback mechanism, and after several iterations, it reaches the SNc, which sends dopamine neuron outputs to the DLS. PFC regulates this serial loop circuit by sending powerful glutamatergic neurons to both the NAc and DLS (Haber et al., 2000; Ikemoto, 2007). Indeed, during the transition to compulsive drug use, the PFC gradually loses control over this serial loop circuit. In the competition between goal-directed and habit-directed behaviors, control is gradually shifted to the habit system dominated by the DLS. This shift in control dynamics is a crucial aspect of the development of compulsive drug-seeking behavior.

## 8 Preliminarily exploring the synergistic dynamics of personality traits, social factors, and neurobiology

In the preceding sections, we have elaborated on the potential driving roles of Personality Traits, Social Factors, and Neurobiology in the individual’s progression toward addictive behavior. However, as mentioned, these three factors do not operate in isolation; rather, they interact in intricate ways to propel individuals toward compulsive drug-seeking behaviors. Therefore, a preliminary discussion on the complex interactions among these three factors would aid in a better understanding of the driving forces behind individual progression toward addiction.

Although individuals with high novelty-seeking and impulsivity tendencies may exhibit similar behavioral characteristics, not all of them will manifest susceptibility to addiction. This suggests the existence of other significant driving factors. It is noteworthy that environmental pressures, especially negative experiences in early life, may promote individuals to display externalizing disorders, characterized by elevated novelty-seeking and impulsivity. These negative experiences include early maternal deprivation, establishment of insecure social relationships, and familial adversities.

Overall, social environmental factors can influence individual behavioral traits, and this influence may be enduring and stable. However, current research on the effects of early environmental pressures on these behaviors is predominantly conducted using rodent models, despite considerable similarities to human-related research and its implications for addiction susceptibility. Considering the ecological validity and translational potential of research findings, we recommend that future studies involving human subjects may appropriately consider the following methods: (1) Large-scale cross-lagged panel studies: by measuring the impact of stress and negative experiences, such as social factors, on individual novelty-seeking and impulsivity tendencies at different time points, explore the causal relationship between these factors and analyze their cross-lagged effects to determine the direction of the influence of these social factors on individual novelty-seeking and impulsivity traits; (2) machine learning: High-Dimensional Data Analysis: Machine learning algorithms are capable of processing large, high-dimensional datasets from both rodent and human studies, enabling the identification of underlying patterns that may not be discernible through traditional statistical methods. For instance, machine learning can uncover specific gene expression profiles, neural activity patterns, or behavioral markers that are consistent across species. Cross-Species Feature Mapping: by comparing features across species, machine learning can identify conserved biological processes or neural circuits that are relevant to both rodents and humans. This facilitates the identification of rodent behaviors or physiological responses that are most predictive of human outcomes. Simulations and Outcome Predictions: Machine learning allows for the simulation of how interventions or treatments effective in rodents might translate to humans. By integrating data from rodent models with human epidemiological data, these models can predict potential human outcomes, enabling more informed decision-making in the early stages of clinical trials.

The inclination toward novelty-seeking, which delineates an individual’s propensity for heightened behavioral responses to novel stimuli and potential rewards, may signify an inherent neurobiological predisposition toward sensitization to cues (Budygin et al., 2020; Koshy Cherian et al., 2017). For instance, neural substrates involving DA neurons encoding circuitry originating from the VTA and SNc, projecting to forebrain targets such as the NAc, are implicated. Previous studies have also indicated that novelty-seeking may engage shared neural pathways with addiction (Kutlu et al., 2022; Rohan et al., 2021).

Furthermore, the lack of planning or goal-directedness within the trait of impulsivity may link habits with compulsive drug-seeking behaviors. It is precisely this top-down mediated lack of control that renders abandonment of drug use and restoration of goal-directedness challenging. Impulsivity thus emerges as a potent driving force for individuals transitioning into a habitual dominance of compulsive drug-seeking patterns (Lüscher et al., 2020). These behavioral characteristics are associated with corresponding neurobiological substrates and may further shape and strengthen the neural connections associated with them. As mentioned earlier, social environmental factors may influence individual novelty-seeking and impulsivity traits, and this influence similarly extends to neurobiological factors. For instance, the hypothalamic–pituitary–adrenal (HPA) axis, widely implicated in stress and stress-related responses, is also believed to directly influence behaviors related to sensation seeking and goal-directed behavior (Tomie et al., 2004).

Therefore, in future research, investigators may consider employing methods such as biomarkers and neuroimaging techniques. These tools, utilized to measure biological and neural system responses, can offer insights into the impact of social environmental factors such as early maternal deprivation, establishment of insecure social relationships, and familial adversities on the neurobiology of addiction.

## 9 Concluding summary

In fact, in the fifth edition of the Diagnostic and Statistical Manual of Mental Disorders (DSM-5) by the American Psychiatric Association, there has been a gradual shift away from emphasizing drug dependence in the diagnostic criteria for substance use disorders. Instead, a specific emphasis has been placed on the compulsive features exhibited by individuals with addiction. This includes behaviors such as dedicating substantial time and financial resources to drug-seeking, neglecting fundamental activities (such as essential employment and basic social interactions), and persisting in drug intake despite experiencing negative physical and psychological consequences (American Psychiatric Association, 2013).

This underscores the increasing support for compulsive drug-seeking behavior as a core symptom in drug addiction, highlighting the significance of understanding the transition of individual behaviors into compulsive drug use. In particular: (1) before the manifestation of compulsive drug-seeking behavior, individuals retain flexibility in adjusting their expectations of goal values. However, following the onset of compulsive drug-seeking behavior, individuals exhibit diminished sensitivity to the devaluation of drugs. As discussed earlier, this observation may suggest the existence of distinct stages of sensitization and habituation during addiction; (2) precisely delineating these stages in the transition to addiction may aid in comprehending the complex and rich neuroscientific findings. Numerous neural circuits and neurotransmitters have been implicated in addiction, and distinguishing these results based on the temporal dimension of addiction progression may provide greater clarity.

Additionally, this review provides important insights into the development of more targeted intervention strategies for substance abuse. From a behavioral perspective, attention training might be beneficial in addressing sustained attention deficits, which could be fundamental to capturing cues with high incentive value. From a social factors standpoint, minimizing prolonged exposure to high-stress environments could further reduce the likelihood of externalizing disorders, thereby enhancing appropriate behavioral inhibition. From a neurobiological perspective, therapeutic approaches focusing on the modulation of dopamine pathways, particularly within the nucleus accumbens and its interactions with the prefrontal cortex and dorsal striatum, could aim to restore the balance between goal-directed and habitual behaviors. This may involve the use of pharmacological agents that modulate dopamine release or uptake, as well as interventions that enhance cortical control over subcortical structures to prevent the dominance of habit-driven behaviors. Moreover, targeting glutamatergic connections within these circuits might offer additional avenues for reducing the compulsivity associated with drug addiction. These perspectives provide valuable insights for the clinical development of prevention or intervention strategies for substance abuse.

In summary, our preceding discourse systematically reviewed the distinct phenotypes of Sign-Tracker (ST) and Goal-Tracker (GT) within the Pavlovian Conditioned Approach (PCA) paradigm. The emphasis was placed on highlighting that the observed differences between these phenotypes may unveil inherent susceptibility traits in addiction. Furthermore, our exploration revealed that several features associated with the ST phenotype may be intricately linked to compulsive drug use in addiction. Notably, these features include suboptimal top-down behavioral control, attentional deficits, and impulsive behavioral traits (Tomie, 2018). These traits are not limited to animal models; similar or analogous traits have been found in human studies, suggesting the potential for translating individual differences identified in animal models to human research (Colaizzi et al., 2020; Garofalo and di Pellegrino, 2015).

Therefore, we summarized the role of personality traits, specifically novelty seeking and impulsivity, in addiction. Overall, individuals with these two traits exhibit core features of the ST phenotype: poor attention and difficulties in behavioral inhibition. Furthermore, we ascertain that the behavior of ST may be associated with early environmental factors and events, such as early stress and attachment relationships. This observation potentially unveils the societal driving factors contributing to the development of compulsive drug use in humans.

Finally, we summarized the current understanding of the neurobiological mechanisms underlying compulsive drug use. These studies suggest that the transition from controlled drug use to uncontrolled, compulsive drug-seeking behavior is rooted in habitual behavior (Barker et al., 2015; Feil et al., 2010; Furlong et al., 2014). These susceptibility traits for addiction are likely influenced by a complex interplay of genetic, epigenetic, neurobiological, and environmental factors. Numerous studies have demonstrated the significant role of genetic associations in addiction and related phenotypes, which underscores the need to further explore these genetic influences. Future research should aim to integrate genetic studies, epigenetic analyses, longitudinal tracking, and cross-addiction spectrum studies to better understand the predictive utility of these susceptibility traits and to disentangle the pharmacological effects of addictive substances. This comprehensive approach will help elucidate the shared and distinct pathways contributing to addiction, ultimately advancing the field’s understanding of the multifaceted nature of drug dependence.
